# Comparative study of Interleukin-18 (IL-18) serum levels in adult onset Still’s disease (AOSD) and systemic onset juvenile idiopathic arthritis (sJIA) and its use as a biomarker for diagnosis and evaluation of disease activity

**DOI:** 10.1186/s41927-019-0053-z

**Published:** 2019-02-28

**Authors:** Holger Kudela, Susanne Drynda, Anke Lux, Gerd Horneff, Joern Kekow

**Affiliations:** 1 Clinic of Rheumatology, University of Magdeburg, Sophie-von-Boetticher-Strasse 1, 39245 Vogelsang-Gommern, Germany; 2 Institute for Biometry and Medical Informatics, University of Magdeburg, Leipziger Strasse 44, 39120 Magdeburg, Germany; 3 Department of General Pediatrics, Asklepios Clinic Sankt Augustin, Arnold-Janssen Strasse 29, 53757 Sankt Augustin, Germany; 4 Department of Pediatric and Adolescents medicine, Medical Faculty, University Hospital of Cologne, Cologne, Germany

**Keywords:** Adult onset Still’s disease (AOSD), Systemic onset juvenile arthritis (sJIA), Interleukin-18 (IL-18), Disease activity

## Abstract

**Background:**

Signs and symptoms establish the diagnosis of adult onset Still’s disease (AOSD) as well as of systemic onset juvenile idiopathic arthritis (sJIA). The published data regarding the importance of IL-18 as a marker for diagnosis and disease activity so far are conflicting. The aim of this study was to clarify the role of IL-18 as a diagnostic and disease activity marker in AOSD and sJIA.

**Methods:**

Thirty adult patients diagnosed with AOSD and twenty children diagnosed with sJIA were included in the study. Clinical and laboratory data were obtained retrospectively for each patient visit whenever IL-18 serum levels were determined. IL-18 levels were determined by ELISA. Sixty-five adults and twenty-three children presenting with fever and/or arthritis who did not meet the criteria for a diagnosis of AOSD or sJIA served as comparison groups. Rau’s criteria and CRP values were used to evaluate disease activity.

**Results:**

IL-18 levels were significantly elevated in patients with active AOSD compared to AOSD patients in remission and to the comparison group with a median of 16,327 pg/ml, 470 pg/ml, and 368 pg/ml, respectively (*p* < 0.001). Analogous to AOSD in active sJIA, the median IL-18 serum level was significantly higher with 21,512 pg/ml than in the comparison group with 2580 pg/ml (*p* < 0.001).

At our cut-off point of 5000 pg/ml, the calculated specificity of IL-18 to establish the diagnosis of AOSD was 96.9%, and the sensitivity 63.3% (AUC = 0.870, p < 0.001). For the diagnosis of sJIA, a cut-off value of 10,000 pg/ml was chosen with a specificity of 100% and a sensitivity of 60% (AUC = 0.774, *p* = 0.003). At a cut-off value of 5000 pg/ml, the specificity was 62% and the sensitivity 65%.

**Conclusions:**

This study gives further evidence to earlier publications of elevated IL-18 serum levels in active AOSD and sJIA, with up to 1000-fold higher concentrations compared to other rheumatic diseases. A clear association of IL-18 serum levels with disease activity in AOSD was found. The results support the use of IL-18 as an important biomarker in AOSD and sJIA.

**Electronic supplementary material:**

The online version of this article (10.1186/s41927-019-0053-z) contains supplementary material, which is available to authorized users.

## Background

Adult onset Still’s disease (AOSD) and systemic onset juvenile idiopathic arthritis (sJIA) are both considered to be multifactorial autoinflammatory diseases with a predominant activation of the innate immune system [1–4]. Autoinflammatory diseases are characterized as inflammatory diseases without showing autoantibodies or antigen-specific autoreactive T-lymphocytes. The primarily effector cells are monocytes and neutrophils [2, 5]. Despite differences, AOSD and sJIA most likely represent the same disease at different stages on an age continuum. Nevertheless, further research is needed to prove the relationship between AOSD and sJIA [1, 6]. Both are rare diseases with an incidence of 0.16–0.4/100,000 for AOSD [7–9] and 0.4–0.9/100,000 in children younger than sixteen years with sJIA [10]. Clinical symptoms of both diseases are quite similar [11–13]. Cardinal symptoms are quotidian fever, arthralgia/arthritis and a salmon colored evanescent rash. Other common symptoms that can occur are sore throat, lymphadenopathy or splenomegaly, hepatomegaly, serositis, and myalgia. Despite macrophage activation syndrome (MAS), life threatening complications are rare [14–16]. Laboratory abnormalities include high erythrocyte sedimentation rates (ESR), elevated CRP, leukocytosis with neutrophilia, thrombocytosis, elevated liver enzymes, elevated ferritin and anemia [14, 15]. The diagnosis of the diseases is based on clinical characteristics. Other differential diagnoses like viral and bacterial infections, malignancies, vasculitis, connective tissue diseases and other autoinflammatory and rheumatic diseases have to be ruled out [14, 17, 18]. To help establish the diagnosis of AOSD, several sets of classification criteria have been developed [19–22]. Best established ar the Yamaguchi-Criteria [21]. In sJIA, the ILAR (International League of Associations for Rheumatology) set of classification criteria [23] is widely used. Until now there is no established specific biomarker for AOSD or sJIA but a couple of cytokines are markedly elevated in both diseases. More recently most attention has been given to Interleukin-18 (IL-18) and the S100 proteins. In several studies IL-18 has been described as a potential biomarker to support the diagnosis of AOSD or sJIA but, so far, the data for use as a marker for disease activity are conflicting [24–36]. The aim of this study was to further elucidate the role of IL-18 as a diagnostic marker and its importance as a measure of disease activity in AOSD and sJIA. Furthermore, the study compared changes of IL-18 levels between AOSD and sJIA. Results of this study have been presented at the 2014 ACR/ARHP Annual Meeting in Boston (Abstract 834) [37].

## Methods

In case of suspected or diagnosed AOSD our physicians have been measuring interleukin 18 levels in clinical practice since 2007. In this noninterventional retrospective study, data from 237 samples taken from 161 patients were available for further analysis. Complete clinical data were available for thirty adult patients diagnosed with AOSD and twenty children diagnosed with sJIA. Clinical characteristics are depicted in Tables 1 and 2. Table 1 also shows a non-AOSD control group with complete clinical data. This group consisted of 65 adults with 48 different diagnoses and fever episodes in their history (for further details see Additional file 1: Table S1). A non-sJIA control group (see also Table 2) consisted of 23 children with 22 different rheumatic and/or inflammatory diseases (for further details see Additional file 1: Table S2). Classification of AOSD and sJIA was made according to Yamaguchi-Criteria [21] for the adults and according to ILAR-Criteria [23] for the children. For evaluation of the disease activity, a modified Pouchot-Activity Score [38], the Rau-Score [39], was used. It consists of 12 typical disease parameters. Each one accounts for one point. The higher the score, the higher is the disease activity. The following parameters are included: fever, evanescent rash, sore throat, arthritis, myalgia, pleuritis, pericarditis, pneumonitis, lymphadenopathy, hepatomegaly or pathological liver function tests, leucocyte count > 15,000/μl, and serum ferritin > 3000 μg/l. Three different disease states were defined: active disease, partial remission and remission. Rau’s Score [39] was used for adults and children. The use of the Juvenile Arthritis Disease Activity Score (JADAS) [40] or the Wallace criteria [41] as measure for inactive disease in the children’s cohort was discarded since there was no data available for the patient or parent global assessment or the physician global assessment. Furthermore, the JADAS [40] is of limited use since one fourth of the score is based on an active joint count, knowing that in sJIA only few joints if at all are affected. Disease states are defined according to Table 3 using the Rau’s Score [42] and CRP.

**Table 1:** Clinical and laboratory characteristics of adult cohort

	AOSD	Control group
Number	30	65
Mean Age (SD; Range)	39 (±16.9; 19–72)	50.9 (±13.5; 23–81)
Sex (male:female)	4:1	1.5:1
Swollen joints per patient* *n* (Range)	0 (0–16)	0 (0–23)
Patients with swollen joints *n* (%)	12 (40)	17 (26)
Tender joints per patient* *n* (Range)	1 (0–24)	0 (0–36)
Patients with tender joints *n* (%)	17 (57)	31 (48)
Arthralgia *n* (%)	21 (70)	40 (62)
Erosive arthritis n	3	3
Fever *n* (%)	20 (66)	31 (48)
Rash *n* (%)	13 (43)	4 (6,2)
Pharyngitis/sore throat *n* (%)	8 (27)	4 (6,2)
Splenomegaly *n* (%)	6 (20)	3 (4,6)
Lymphadenopathy *n* (%)	2 (6.7)	1 (1,5)
Serositis (Pleuritis/Pericarditis/Peritonitis) *n* (%)	3 (10)	4 (6,2)
Prednisolone Therapy at IL-18 determination *n* (%)	15 (50)	24 (37)
DMARD Therapy	11 (37)	16 (25)
WBC > 10,000/ml *n* (%)	11 (37)	22 (34)
WBC Gpt/ml (SD; Range)	11.7 (± 8.6; 3.9–46.5)	9.4 (± 4.1; 2.3–21.5)
Neutrophils > 80% *n* (%)	11 (37)	13 (20)
ANA > 1:160 *n* (%)	3 (10)	6 (0.09)
Elevated ALAT or ASAT *n* (%)	9 (30)	17 (26)
ALAT μmol/ls (SD)	0.83 (±0.96; 0.23–4.99)	0.73 (±0.63; 0.1–4.8)
RF positive *n* (%)	1 (0.03)	13 (20)
CRP mg/l (SD; Range)	76.3 (±71.5; < 5–231.7)	45.5 (±62.3; < 5–220)
ESR mm/1 h (SD; Range)	45.0 (±30.1; 1–95)	46.7 (±37.5; 2–150)
RBC Tpt/l (SD; Range)	4.29 (±0.57; 3.18–5.29)	4.37 (±0.95; 2.25–9.6)
Hemoglobin mmol/l (SD; Range)	7.7 (±1.1; 5.6–10)	7.8 (±1.2; 4.3–10)
Hematocrit % (SD; Range)	0.37 (±0.05;0.27–0.46)	0.38 (±0.05;0.21–0.48)
Platelets Gpt/l (SD; Range)	310 (±115.3; 126–624)	326 (±115.4; 83–621)
IL-18 pg/ml* (Range)	10,425 (100–408,000)	355 (87.2–6600)

*Median; *SD* Standard Deviation, *n* number, *DMARD* disease modifying antirheumatic drug, *WBC* white blood cell count, *ANA* antinuclear antibodies, *ALT* alanine transaminase, *AST* aspartate transaminase, *RF* rheumatoid factor, *CRP* C-reactive protein, *ESR* erythrocyte sedimentation rate, *RBC* red blood cell count

**Table 2:** Clinical and laboratory characteristics of children’s cohort

	sJIA	Non-sJIA comparison group in children
Number	20	23
Mean age in years (SD; Range)	9.6 (±5.5; 2–17)	7.8 (±5.1; 11 Mon.-17)
Sex (male:female)	1.2:1	1:1.1
Swollen joints per patient* *n* (Range)	0.5 (0–10)	0 (0–8)
Patients with swollen joints *n* (%)	10 (50)	4 (12.5)
Tender joints per patient* n (Range)	1 (0–10)	0 (0–8)
Patients with tender joints *n* (%)	12 (60)	5 (15.6)
Arthralgia *n* (%)	14 (70)	9 (39.1)
Erosive Arthritis *n*	0	0
Fever *n* (%)	16 (80)	16 (69.6)
Rash *n* (%)	12 (60)	3 (13)
Pharyngitis/sore throat *n* (%)	4 (20)	1 (4.3)
Splenomegaly *n* (%)	3 (15)	2 (8.7)
Lymphadenopathy *n* (%)	0 (0)	4 (17.4)
Serositis (Pleuritis/Pericarditis/Peritonitis) *n* (%)	2 (10)	3 (13)
Prednisolone Therapy at IL-18 determination *n* (%)	5 (25)	4 (17.4)
DMARD Therapy	2 (10)	2 (8.7)
WBC > 10,000/ml *n* (%)	16 (80)	11 (47.8)
WBC Gpt/ml (SD; Range)	15.1 (± 8.3; 5.6–37.7)	12.8 (± 8.4; 3.6–32.8)
Neutrophils > 80% *n* (%)	8 (40)	5 (21.7)
ANA > 1:160 *n* (%)	0 (0). 6 x not examined	2 (8.7) 8 x not examined
Elevated ALT or AST *n* (%)	1 (5)	2 (8.7)
ALAT μmol/ls (SD)	0.3 (±0.20; 0.07–0.72)	0.35 (±0.21; 0.1–0.87)
RF positive *n* (%)	2 (7 x of 20 not examined)	1 (8 x of 23 not examined)
CRP mg/l (SD; Range)	0 (8 x of 20 not examined)	0 (13 x of 23 not examined)
ESR mm/1 h (SD; Range)	115.1 (±85.4; < 5–398.8)	70.6 (±74.6; < 5–253.4)
WBC > 10,000/ml *n* (%)	70.0 (±48.9; 2–147; 12 x not examined)	51.1 (±42.4; 5–125; 16 x not examined)
RBC Tpt/l (SD; Range)	4.46 (±0.53; 3.4–5.53)	4.33 (±0.51; 3.15–5.47)
Hemoglobin mmol/l (SD; Range)	8.3 (±2.1; 6–12)	8.4 (±2.46; 5.2–13.8)
Hematocrit % (SD; Range)	0.35 (±0.04;0.29–0.46)	0.34 (±0.04;0.26–0.42)
Platelets Gpt/l (SD; Range)	409.2 (±178.8; 166–687)	360.4 (±124.5; 183–660)
IL-18 pg/ml* (Range)	14,732.5 (215–372,850)	2580 (346.2–141,650)

*Median; *SD* Standard Deviation, *n* number, *DMARD* disease modifying antirheumatic drug, *WBC* white blood cell count, *ANA* antinuclear antibodies, *ALT* alanine transaminase, *AST* aspartate transaminase, *RF* rheumatoid factor, *CRP* C-reactive protein, *ESR* erythrocyte sedimentation rate, *RBC* red blood cell count

**Table 3:** Definition of disease states in AOSD and sJIA

	CRP ≥ 2 x ULN	CRP < 2 x ULN
Rau Score ≥ 2	Active Disease	Partial Remission
Rau Score < 2	Partial Remission	Remission

*CRP* C-reactive protein, *ULN* Upper Limit of Normal

IL-18 serum concentrations were determined with an IL-18 Sandwich ELISA (MBL: Medical & Biological Laboratories, Nagoya, Japan) according to the manufacturer’s instructions.

For statistical analysis the software IBM SPSS Statistics (V 21.0) was used. Data were presented as mean, standard deviation (SD), median, range, and the 95% confidence interval. Tests applied were the Wilcoxon signed rank test, Kruskal-Wallis H test, Mann-Whitney U test, and the Pearson correlation. Two-tailed *P* values less than 0.05 were considered significant. Area under the receiver operating characteristic curve (ROC-AUC) was used to evaluate the diagnostic value of the IL-18 serum level for AOSD or sJIA.

## Results

### AOSD

Of the 30 patients diagnosed with AOSD, 20 met the Yamaguchi criteria. Two did not meet the criteria only because of positive ANA titer. For four patients, the Yamaguchi criteria could not be applied, because of limited clinical records for the time of disease onset. Four patients with AOSD did not meet the Yamaguchi classification criteria. Only five patients would have met the ILAR criteria, four of them met the Yamaguchi classification criteria as well. The definite diagnosis of an AOSD in all cases was made by at least two experienced rheumatologists, were checked by the investigators and retained when clinically sound. During the observation period 20 of the 30 AOSD patients received a DMARD (Disease-modifying antirheumatic drug) therapy, eleven patients received methotrexate, ten anakinra, as well in monotherapy as in direct combination or in combination with other drugs (anakinra+leflunomide, methotrexate+etanercept, methotrexate+cyclosprine). Two thirds of the patients had a glucocorticoid therapy.

### AOSD patients with active disease at first IL-18 determination

Twelve patients with active disease at their first visit had at least one IL-18 follow-up. One of the 12 patients refused any treatment, had persistent active disease at the last follow- up and was not part of the further analysis. Nine patients were in remission and two were in partial remission after 27.5 (±26.4) months. CRP and IL-18 serum levels as well as the Rau-Score decreased significantly in the course of treatment compared to the first visit with IL-18 serum determination in active disease (Wilcoxon-signed-rank test *p* < 0.001). For further details see Table 4.

**Table 4:** Characteristics of AOSD patients with initially active disease and follow up at first and last visit

Parameter	First IL-18 determination	Last IL-18 determination
Number	11	11
Age in years (SD; Range)	38 (±16.0; 19–63)	40.4 (±16.0; 20–64)
Time after first IL-18 determination in months (SD; Range)	0	27.5 (±26.4; 3–67)
Rau Score (SD; Range)	4.18 (±1.47; 2–6)	0.36 (±67; 0–2)
CRP mg/l (SD; Range)	104.4 (±68.4; 16.2–208)	7.8 (±8.3; < 5–32.7)
ESR mm/1 h (SD; Range)	51 (±23; 11–82)	14.5 (±13.4; 2–47)
IL-18 pg/ml (Range)*	12,500 (850–408,000)	402 (20–7560)
WBC Gpt/ml (SD; Range)	15.0 (± 11.6; 5.4–46.5)	7.3 (± 1.78; 4.9–9.7)

*Median; *SD* Standard Deviation, *CRP* C-reactive protein, *ESR* erythrocyte sedimentation rate, *WBC* white blood cell count

### Clinical and laboratory findings

**Fig. 1:**
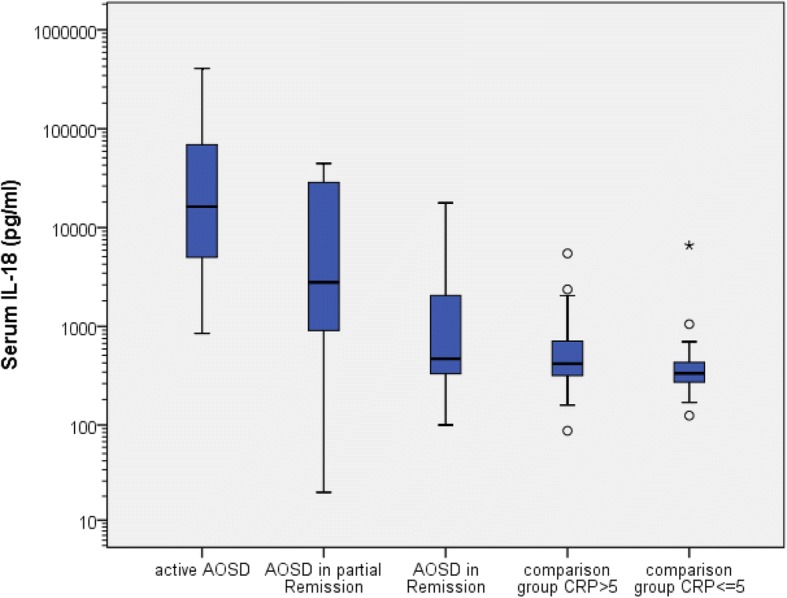
Box-and-whisker plot of IL-18 serum levels in adult cohort

**Fig. 2:**
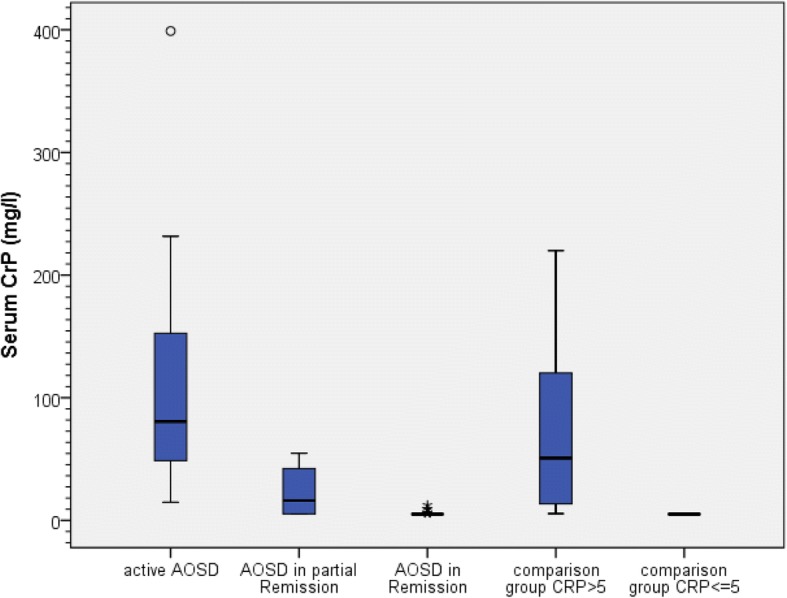
Box-and-whisker plot of CRP serum levels in adult cohort

IL-18 was determined in 158 serum samples from 95 patients (30 AOSD, 65 controls). Very high levels of IL-18 > 5000 pg/ml were only seen in active AOSD except in 2 other patients, one with chronic lymphatic leukemia (6600 pg/ml) and one with sepsis (5476 pg/ml). IL-18 serum levels had a positive correlation with CRP levels (r = 0.563, *p* < 0.001), Rau Score (r = 0.744; *p* < 0.001) and ferritin (r = 0.551, *p* < 0.001) only in patients with AOSD. For analysis a Pearson correlation adapted with a logarithmic transformation was used. The control group was divided into a group with elevated CRP (> 5 mg/l) and normal CRP (< 5 mg/l). Figures 1 and 2 show box-and-whisker plots of IL-18 and CRP serum levels for the different patient groups. IL-18 serum levels were significantly elevated in active AOSD 70,821 ± 108,851 pg/ml (median 16,327 pg/ml) compared to all other groups (*p* < 0.001) except AOSD in partial remission (*p* = 0.766), depicted in Fig. 1. In contrast, no difference was found in CRP levels (Fig. 2) of the active AOSD and the comparison group with CRP > 5 (*p* = 1.000), whereas compared with the other groups the CRP levels were significantly elevated (*p* < 0.001–0.033). In addition, the Rau Score in active AOSD was significantly elevated compared to AOSD in partial remission (*p* = 0.009) and to AOSD in remission (*p* < 0.001). ROC-AUC analysis for the IL-18 serum level between AOSD patients and the comparison group was 0.870 and significantly diagnostic for AOSD (*p* < 0.001; 95% Confidence Interval 0.775–0.965). At a cut-off point of ≥5000 pg/ml, sensitivity for diagnosis of AOSD was 63% and specificity 97%. At a cut-off point for the IL-18 serum level of 832.5 pg/ml, corresponding to the highest value for the Youden-Index (0.68), sensitivity was 80% and specificity 81.5%.

#### sJIA

**Fig. 3:**
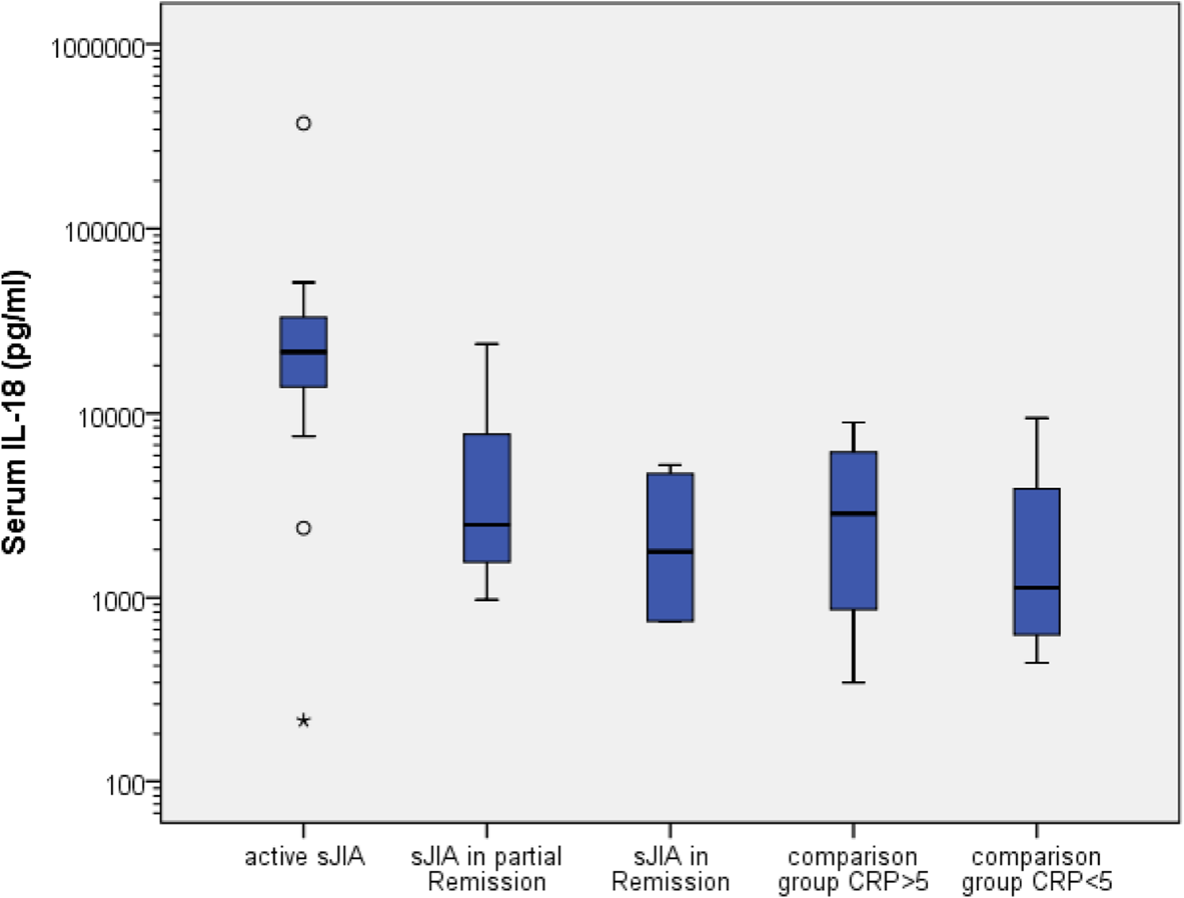
Box-and-whisker plots for IL-18 serum levels in children’s cohort

**Fig. 4:**
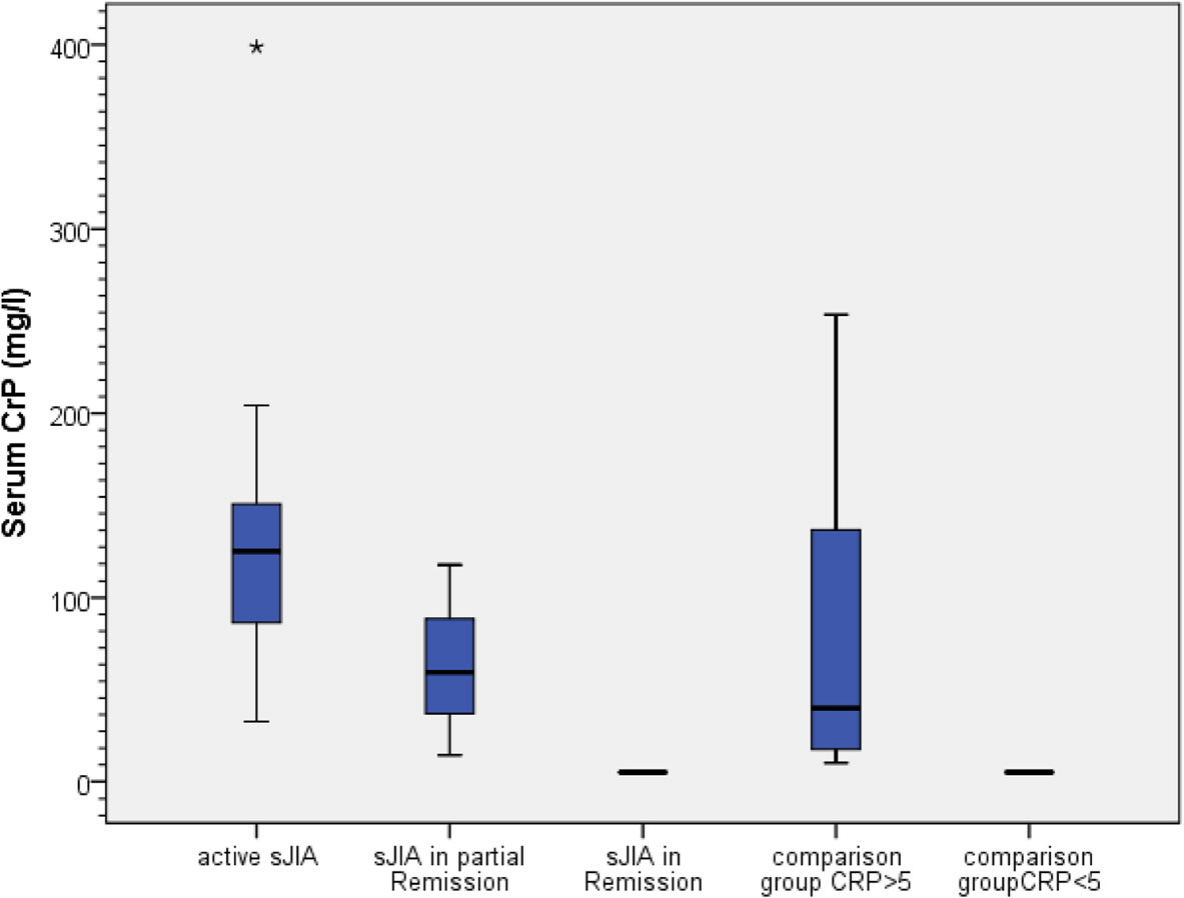
Box-and-whisker plots for serum CRP in children’s cohort

Twenty patients with sJIA were included in the study. The definite diagnosis of sJIA in all cases was made by at least two experienced pediatric rheumatologists, were checked by the investigators and retained when clinically sound. Eleven met the ILAR Criteria, 12 the Yamaguchi Criteria, 10 both and 13 met either one or the other. Five children did not meet any of the criteria. For two patients, not any of the criteria could be applied because of limited clinical records for the time of disease onset. That only 11 of the 20 children enclosed in this study met the ILAR criteria for sJIA reflects findings of Hinze et al. where only 47,8% in the AID registry and only 54,3% of the patients diagnosed with sJIA met the ILAR criteria [43]. As in the German Autoinflammatory Disease (AID) registry cohort and the inception cohort of newly diagnosed patients with JIA (ICON-JIA) [43] 100% of the children with active disease had fever (see Additional file 1: Table S3). During the observation period three patients received methotrexate as DMARD, one methotrexate+adalimumab, one anakinra, and one canakinumab. Sixteen of the twenty children received glucocorticoids during the observed course of the disease. As in AOSD, in sJIA there are very high levels of IL-18 in active sJIA compared to the non-sJIA control group. Here as well sJIA patients with partial or complete remission had notably lower IL-18 serum levels compared to active sJIA, but due to very low sample sizes (sJIA in partial remission *n* = 3; sJIA in remission *n* = 4) *p*-values are not mentioned. Analogous to AOSD, IL-18 serum levels in active sJIA (median = 21,512 pg/ml) were significantly higher than in the non-sJIA control group with elevated CRP (median = 2855 pg/ml; *p* = 0.002) although there was no difference in CRP levels between the two groups (*p* = 1.000). The corresponding box-and-whisker plots are shown in Figs. 3 and 4. ROC-AUC analysis for the IL-18 serum level between sJIA patients and the non-sJIA comparison group was 0.774 and thereby a significant diagnostic measure for sJIA (*p* = 0.003; 95% Confidence Interval 0.621–0.926). At a cut-off point of ≥5000 pg/ml, sensitivity for diagnosis of sJIA was 65% and specificity 62%, at ≥7000 pg/ml 65 and 90.5% and at 10,000 pg/ml 60 and 100%, respectively. At a cut-off point for the IL-18 serum level of 11,473.5 pg/ml, corresponding to the highest value for the Youden-Index (0.61), sensitivity was 61%, specificity 100%.

#### Comparisons between sJIA and AOSD

In addition to the separate evaluation for sJIA and AOSD, further analysis was performed for the active states of either disease in this study. There were no significant differences between the diseases except for age. The mean IL-18 level in the active AOSD group was 90,433 ± 122,123 pg/ml (median 12,500 pg/ml), compared to 42,884 ± 92,045 pg/ml (median 21,025 pg/ml) in the active sJIA group. Further details are shown in Table 5.

**Table 5:** Comparison of clinical details in active sJIA and active AOSD (for definition of disease state see Table 3)

	Age (years)	IL-18 (pg/ml)	CRP (mg/l)	ESR 1st h	WBC (Gpt/l)	Rau Score
Adults						
Number	19	19	19	14	19	19
Mean	39.6	90,433.4	105.3	57.9	14.5	4.0
SD	±17.2	±122,123.1	±65.4	23.2	±9.6	±1.4
Median	40	12,500.0	84.8	59.0	10.6	4.0
Minimum	19	850.0	16.2	11	5.4	2
Maximum	72	408,000.0	231.7	90	46.5	6
Children						
Number	15	15	15	6	15	15
Mean	9.2	42,884.7	140.7	92.2	17.1	3.5
SD	±5.5	±92,045.0	±79.9	±31.5	±8.7	±1.1
Median	8	21,025.0	125.3	91.0	14.1	3
Minimum	3	215.0	73.5	60	5.6	2
Maximum	17	372,850.0	398.8	147	37.7	5

*SD* Standard Deviation, *CRP* C-reactive protein, *ESR* erythrocyte sedimentation rate, *WBC* white blood cell count

## Discussion

AOSD and sJIA are rare diseases. The diagnosis relies on clinical signs and symptoms. The exclusion of other differential diagnoses is crucial [1, 10]. A specific biomarker has not been established to date, even though various cytokines have been studied for this purpose. The first publication of Kawashima et al. in 2001, which described extremely high IL-18 serum levels in AOSD [28], followed a couple of other studies which confirmed these findings for AOSD [24–27, 29, 44–47] and in a smaller proportion as well for sJIA [33–36, 46, 48–50]. The cited references’ authors uniformly acknowledge IL-18 as pivotal in the pathogenesis of the diseases. Extremely high IL-18 serum levels in active disease were also seen in our study and confirm earlier studies. Furthermore, normalization of IL-18 serum levels in remission was observed, analogous to Kawashima et al. [28] and other authors [24, 26, 27, 45, 47, 51], and in contrast to other publications which could not show a decline of IL-18 in remission [25, 29]. Due to the limited follow-up data in the children’s cohort in our study, it can only be assumed that IL-18 serum levels are markedly lower in remission compared to active disease, but is concordant with four other studies [30, 48, 49, 52]. All studies including the present one show a massive elevation of IL-18 serum levels in AOSD compared to other diseases. In active AOSD compared to rheumatoid arthritis, Kawashima et al. showed a 600-fold higher serum concentration of IL-18 [28]. In this study a remarkable 100-fold higher serum concentration of IL-18 was also demonstrated. It is of note that the studies with the same ELISA had similar ranges of IL-18 serum levels. The ranges of the IL-18 serum levels in other ELISAs were different but reacted similarly according to disease state and control group. It is not clear why there are differences in the amount of IL-18 serum levels with otherwise comparable clinical and laboratory parameters and a similar change of IL-18 serum levels corresponding to the disease state. The influence of IL-18 binding protein does not seem to be the cause, since Jung et al. [51] found that despite high serum levels of IL-18 binding protein, there was no significant difference in levels of IL-18 and free IL-18. That ELISAs from different manufacturers measure different forms of IL-18 altered by further processing is a possible explanation but speculation. In this work and the work of Colafrancesco et al. [26], Kim et al. [29], Jung et al. [51] and Priori et al., IL-18 is considered to be a useful diagnostic marker for AOSD. Kim et al. [29] even showed a better specificity and sensitivity of IL-18 for the diagnosis of AOSD than Calprotectin (S100A8/A9 proteins). All studies with a ROC-AUC analysis, except the present one, chose fairly low cut-off points of serum IL-18 levels compared to the massive elevation of serum IL-18 in active AOSD. With higher cut-off values, specificity for the diagnosis of AOSD would be much higher, which would be most beneficial in differentiating AOSD from other inflammatory conditions in daily clinical practice. The area under the curve in Colafrancesco et al.’s work [26] is markedly lower than in this, in Kim et al.’s, Priori et al.’s and Jung et al’s studies [29, 31, 51]. Most likely it is because Colafrancesco et al. used patients with AOSD for the ROC-AUC analysis without taking the disease activity into account. In a study of the same group by Priori et al. [45], which only considered active AOSD compared to sepsis, the area under the curve was almost equal to the study presented here. Specificity differed most likely because of a low cut-off for IL-18. It has to be considered that a different ELISA was used. Regarding the use of IL-18 as an activity marker in AOSD the data is conflicting. Kawashima et al. [28] first described elevated IL-18 serum levels in three patients with active AOSD followed by gradual normalization in disease remission under therapy. Colafrancesco et al. [26], Jung et al. [51] Girard et al. [47] and Priori et al. [45] confirmed these findings and described a significant reduction of IL-18 serum levels in inactive AOSD compared to active AOSD, as is also shown in this study. Kim et al. [29] did not show a significant reduction even though there seems to be a tendency for lower IL-18 levels of AOSD in remission. The main problem often seen in studies with AOSD and IL-18 is a small number of cases with low statistical power. Choi et al. [25] did not show a difference of IL-18 levels in active AOSD compared to inactive AOSD either. The number of cases of AOSD in Choi et al.’s [25] study (14 patients) was even lower, and there was no clear definition of disease activity available. Furthermore, the follow-up period of 3–12 weeks was very short. In our study two patients still showed clearly elevated IL-18 serum levels long after achieving remission (30 months) but showed a gradual normalization as described by Kawashima et al. [28]. Jung et al. discussed that a persisting elevation of IL-18 serum level is an expression of underlying subclinical disease activity [51]. Similar observations were made by Shimizu et al. [49]. Until 2017 the only larger study in sJIA which included a follow-up of IL-18 serum levels was the one done by Jelušić et al. [33]. Results of that study showed a significant reduction of IL-18 serum levels in inactive sJIA compared to active sJIA (*p* < 0.001). Details of the time of follow-up were not stated. IL-18 serum levels in inactive sJIA were still elevated compared to other control groups. In 2017 Brachat et al. [36] published a study on early changes in gene expression and inflammatory proteins in sJIA patients on canakinumab therapy. They also found markedly elevated IL-18 serum levels and a prolonged but statistical not significant reduction of IL-18 serum levels in inactive disease [36]. In our study, having only three patients with follow-up IL-18 determinations in the sJIA cohort, it can only be assumed that a slow but gradual normalization of IL-18 serum levels occurs in remission. The studies on IL-18 as diagnostic marker in sJIA including our own are coherent [33–35, 49, 50]. IL-18 levels in sJIA are always significantly elevated compared to controls. Within the control groups there are no significant differences. All studies used the MBL ELISA. In contrast to all other studies ROC-AUC analysis was only performed in this study. The analysis showed an area under the curve (AUC) of 0.774 (*p* = 0.003; 95% confidence interval 0.621–0.926). This analysis is comparable with a study by Frosch et al. [53] which investigated the use of S100 proteins as diagnostic marker in sJIA. They had an area under the curve (AUC) of 0.747 ± 0.097. The specificity at a cut-off of 9200 ng/ml for S100A8/S100A9 for the diagnosis of sJIA was 95%. In our own work, the specificity for IL-18 for diagnosis of sJIA was 100% at a cut-off for IL-18 of 10,000 pg/ml. It has to be mentioned that Frosch et al. had a much higher number of patients with sJIA (60) and a higher number of patients in the control groups (85 severe systemic infections; 45 leukemias; 18 NOMID). So far there are no head to head studies published comparing IL-18 serum levels and S100 protein serum levels in sJIA. The higher cut-off for IL-18 in sJIA compared to AOSD for a good differentiation from other diseases might be due to different number of patients, or as discussed by Pay et al. for other clinical and laboratory differences of sJIA and AOSD, due to a different reacting immune system of children which have a more naïve immune system [54].

## Conclusions

AOSD and sJIA show very high IL-18 serum levels compared to healthy controls and other rheumatic and inflammatory diseases (up to 1000-fold higher). Higher levels have not been reported in any other disease to date. There is a significant association of serum IL-18 levels with disease activity. IL-18 seems to be a good biomarker to support the diagnosis of AOSD and sJIA. IL-18 serum levels react similarly in AOSD and sJIA and help to monitor disease activity. Serology is already integrated in the 2010 ACR/EULAR classification criteria for rheumatoid arthritis [55] or in the 2012 SLICC criteria for lupus erythematosus [56]. Analogous the data on IL-18 serum levels in AOSD and sJIA encourage the integration of IL-18 serology in a new set of ACR/EULAR classification criteria for AOSD and sJIA.

## Additional file

Additional file 1: **Table S1.** List of main diganoses in adult control group. **Table S2.** List of diseases in children’s control group. **Table S3.** Frequency of symptoms in active sJIA. (DOCX 21 kb)

## Abbreviations

AID: Autoinflammatory Disease
AOSD: Adult onset Still’s disease
AUC: Area under the curve
DMARD: Disease-modifying antirheumatic drug
ICON-JIA: Inception cohort of newly diagnosed patients with JIA
IL-18: Interleukin-18
JADAS: Juvenile Arthritis Disease Activity Score
MAS: Macrophage activation syndrome
NOMID: Neonatal onset multiinflammatory disease
ROC: Receiver operating characteristic curve
sJIA: Systemic onset juvenile idiopathic arthritis
