# Supplementary material for: Comparative study of Interleukin-18 (IL-18) serum levels in adult onset Still’s disease (AOSD) and systemic onset juvenile idiopathic arthritis (sJIA) and its use as a biomarker for diagnosis and evaluation of disease activity
Source: BMC Rheumatol. 2019 Feb 28;3:4. doi: 10.1186/s41927-019-0053-z (PMC6394042; doi:10.1186/s41927-019-0053-z)
Supplement: Supplementary file 1 — Table S1. List of main diganoses in adult control group. Table S2. List of diseases in children’s control group. Table S3. Frequency of symptoms in active sJIA. (DOCX 21 kb) [file 41927_2019_53_MOESM1_ESM.docx]

Table S1 List of main diagnoses adult control group

| Seronegative rheumatoid arthritis (n=4) | Fever of unknown origin (n=6) | Reactive arthritis (n=3) |
| --- | --- | --- |
| Sarkoidosis (n=2) | Healthy (n=2) | Generalized Osteoarthritis (n=2) |
| Chronic lymphatic leukemia | Suspected small vessel vasculitis | Undifferentiated collagenosis |
| State after sarcoidosis | lymphadenopathy | Pericardial effusion |
| ANCA negative vasculitis | State after fever of unknown origin | Suspected exogenous allergic alveolitis |
| subfebrile temperatures of unknown origin | Possible ANCA positive vasculitis | Acute myeloid leukemia |
| FUO, [Brocq-Jaquet neurodermitis](https://de.wiktionary.org/w/index.php?title=Brocq-Jaquet_neurodermitis&action=edit&redlink=1) | ESR und CrP elevation of unknown origin | Suspected reactive arthralgias and myalgias |
| Suspected undifferentiated collagenosis | Morbus Behçet with ocular involvement | Atypical rheumatoid arthritis |
| Aortitis of the abdominal aorta | Sepsis, pulmonary caverns | State after feverish infection |
| Chronic pain syndrome | (suspected) Crohn’s disease | Polymyalgia rheumatica and lower urinary tract infection |
| Osteoarthritis of distal interphalangeal joints of the hands, recurrent fever | Seropositive rheumatoid arthritis | State after liver transplantation due to primary sclerosing cholangitis |
| Seropositive rheumatoid arthritis, suspected myocarditis | Arthralgias of unknow origin | Psoriasis vulgaris |
| Suspected inflammatory rheumatic disease | Systemic lupus erythematosus | eosinophilic fasciitis |
| Myalgias of the lumbar spine | granulomatosis with polyangiitis | Rheumatoid arthritis and osteoporosis |
| Jo-1-Syndrome, lymphoma | T-cell lymphoma |  |
| Undifferentiated polyarthritis | Palindromic rheumatism |  |
| CRP: C-reactive protein; ESR: erythrocyte sedimentation rate; FUO: fever of unknown origin, ANCA: Anti-neutrophil cytoplasmic antibody | | |

Table S2 List of diseases in children’s control group

| Enteropathogenic Escherichia coli (EPEC) enteritis, suspected Still’s disease | PFAPA (periodic fevers with [aphthous stomatitis](https://www.msdmanuals.com/professional/dental-disorders/symptoms-of-dental-and-oral-disorders/recurrent-aphthous-stomatitis), pharyngitis, and adenitis) syndrome | Suspected Still’s disease/protracted infection with lymphadenitis of the neck |
| --- | --- | --- |
| Fever and arthralgias of unknow origin | Protracted fever | Suspected neutrophilic dermatosis |
| Pneumonia with bilateral pleural effusions | Suspected Still’s disease/NOMID | Feverish infection with conjunctivitis |
| Hemophagocytic lymphohistiocytosis | Systemic lupus erythematosus | Suspected PFAPA syndrome |
| Urinary tract infection | TRAPS | State after Kawasaki syndrome |
| JIA (enthesitis-related arthritis) | Familial mediterranean fever | Suspected Kawasaki syndrome/ Still’s disease |
| JIA (possible Still’s disease) | Suspected hereditary fever syndrome/ PFAPA | Suspected Still’s disease/ cytomegalovirus infection, recurrent fever |
| Suspected JIA | Pericardial effusion of unknown origin | Enterococcus-associated nephritis |
| ESR: erythrocyte sedimentation rate; WBC: white blood cell count; FUO: fever of unknown origin; PFAPA syndrome: periodic fevers with [aphthous stomatitis](https://www.msdmanuals.com/professional/dental-disorders/symptoms-of-dental-and-oral-disorders/recurrent-aphthous-stomatitis), pharyngitis, and adenitis syndrome; NOMID: neonatal onset multiinflammatory disease, TRAPS: tumor necrosis factor receptor associated periodic syndrome, JIA: juvenile idiopathic arthritis | | |

Table S3 Frequency of symptoms in active sJIA

| Symptom | number | % |
| --- | --- | --- |
| Fever | 16 | 100 |
| Arthralgias | 14 | 87 |
| Rash | 13 | 81 |
| Arthritis (clinical) | 9 | 56 |
| Arthritis (sonographic) | 9 | 56 |
| Sore throat/Pharyngitis | 4 | 25 |
| Splenomegaly | 3 | 19 |
| Serositis | 2 | 12,5 |

[Table S 1: List of main diagnoses adult control group 1](#_Toc523412868)

[Table S 2: List of diseases in children’s control group 2](#_Toc523412869)

[Table S 3: Frequency of symptoms in active sJIA 2](#_Toc523412870)
